# Cerebellar Continuous Theta Burst Stimulation for Aphasia Rehabilitation: Study Protocol for a Randomized Controlled Trial

**DOI:** 10.3389/fnagi.2022.909733

**Published:** 2022-06-02

**Authors:** Kai Zheng, Mingyun Chen, Ying Shen, Xinlei Xu, Fanglan Gao, Guilan Huang, Yingying Ji, Bin Su, Da Song, Hui Fang, Peng Liu, Caili Ren

**Affiliations:** ^1^Department of Rehabilitation Medicine, The First Affiliated Hospital, Sun Yat-sen University, Guangzhou, China; ^2^Department of Neurorehabilitation, Wuxi Tongren Rehabilitation Hospital, Wuxi, China; ^3^The Affiliated Wuxi Mental Health Center of Nanjing Medical University, Wuxi, China; ^4^Rehabilitation Medicine Center, The First Affiliated Hospital of Nanjing Medical University, Nanjing, China

**Keywords:** study protocol, aphasia, continuous theta burst stimulation, cerebellum, randomized controlled trial (RCT)

## Abstract

**Background:**

Language recovery is limited in moderate to severe post-stroke aphasia patients. Repetitive transcranial magnetic stimulation (rTMS) has emerged as a promising tool in improving language dysfunctions caused by post-stroke aphasia, but the treatment outcome is as yet mixed. Considerable evidence has demonstrated the essential involvement of the cerebellum in a variety of language functions, suggesting that it may be a potential stimulation target of TMS for the treatment of post-stroke aphasia. Theta burst stimulation (TBS) is a specific pattern of rTMS with shorter stimulation times and better therapeutic effects. The effect of continuous TBS (cTBS) on the cerebellum in patients with aphasia with chronic stroke needs further exploration.

**Methods:**

In this randomized, sham-controlled clinical trial, patients (*n* = 40) with chronic post-stroke aphasia received 10 sessions of real cTBS (*n* = 20) or sham cTBS (*n* = 20) over the right cerebellar Crus I+ a 30-min speech-language therapy. The Western Aphasia Battery (WAB) serves as the primary measure of the treatment outcome. The secondary outcome measures include the Boston Diagnostic Aphasia Examination, Boston Naming Test and speech acoustic parameters. Resting-state fMRI data were also obtained to examine treatment-induced changes in functional connectivity of the cerebro-cerebellar network. These outcome measures are assessed before, immediately after, and 12 weeks after cerebellar cTBS intervention.

**Discussion:**

This protocol holds promise that cerebellar cTBS is a potential strategy to improve language functions in chronic post-stroke aphasia. The resting-state fMRI may explore the neural mechanism underlying the aphasia rehabilitation with cerebellar cTBS.

## Introduction

Aphasia is an acquired language disorder caused by ischemic or hemorrhagic stroke (Engelter et al., 2006), characterized by impairments in verbal fluency, language comprehension, word repetition, and picture naming as well as reading/writing skills. Approximately, one-third of post-stroke patients develops aphasia (Engelter et al., 2006), which persists in approximate 20% of long-term stroke survivors (Berthier, 2005). Patients with post-stroke aphasia suffer from decreased quality of life, limited independence, and substantial long-term disability (Cruice et al., 2011; Ellis et al., 2012). To date, speech-language therapy (SLT) is considered as the standard protocol for the treatment of post-stroke aphasia and produce beneficial effects on language functions, but the effect size is somewhat small that results in modest improvements (Brady et al., 2012). Therefore, it is important to develop other therapeutic approaches to improve the effectiveness of SLT for aphasia treatment.

In recent years, repetitive transcranial magnetic stimulation (rTMS) has emerged as a promising non-invasive neuromodulation method to augment the treatment of post-stroke non-fluent aphasia (Hartwigsen and Saur, 2017; Fisicaro et al., 2019; Ren et al., 2019). Generally, low-frequency rTMS (≤1 Hz) is considered to decrease cortical excitability, while high-frequency rTMS (≥5 Hz) produces the opposite effect (Chen and Seitz, 2001). The majority of recent studies have shown naming improvements in post-stroke non-fluent aphasia by delivering inhibitory low-frequency rTMS over the intact right inferior frontal gyrus (IFG) (the right hemisphere homolog of Broca’s area) (Hara et al., 2015; Harvey et al., 2017; Ren et al., 2019; Kim et al., 2020). Similar beneficial effects on post-stroke aphasia were also observed when excitatory high-frequency rTMS was applied over the left perilesional cortex (Broca’s area) or right intact IFG (Hara et al., 2017; Hu et al., 2018). Alternatively, applying rTMS over other cortical regions is also beneficial for post-stroke aphasia, as evidenced by improvement in speech perception and auditory comprehension following low-frequency rTMS over the right posterior superior temporal gyrus (pSTG) (Kawamura et al., 2019; Ren et al., 2019). Nevertheless, it is noteworthy that the evidence for beneficial effects of rTMS on post-stroke aphasia is not unequivocal (Breining and Sebastian, 2020). Some studies reported null results when low-frequency rTMS was applied over the right hemisphere, showing that rTMS did not add to the effect of SLT on post-stroke aphasia (Santos et al., 2017; Heikkinen et al., 2018). Among many possible resources of the inconsistencies across the studies investigating the efficacy of rTMS for post-stroke aphasia are type of stimulation modality (inhibitory or excitatory), laterality of brain hemisphere (left or right), and choice of stimulation target (Broca’s area, STG, etc.) (Breining and Sebastian, 2020). Particularly, the optimal protocol of rTMS over the right hemisphere remains controversial for aphasia rehabilitation (Anglade et al., 2014), which is due to the limited knowledge about the causal links between cortical brain regions and language functions. Accordingly, developing a novel stimulation protocol with other potential targets that are significantly involved in language processing is important for improving effectiveness of TMS in augmenting the treatment of post-stroke aphasia.

The present study protocol proposes a research protocol that stimulates the right cerebellum with TMS to augment SLT for patients with post-stroke aphasia. Multiple lines of evidence have demonstrated significant contributions of the right cerebellum to a variety of language functions, including word retrieval and generation, verbal working memory, language learning, and semantic processing (Gordon, 1996; Desmond and Fiez, 1998; Murdoch, 2010; Stoodley, 2012; Mariën and Borgatti, 2018). Clinical evidence has shown deficits in a variety of language tasks as a result of damage to the right cerebellum (Marien et al., 1996, 2001; Silveri et al., 1998). Moreover, a growing body of empirical and clinical studies using transcranial direct current stimulation (tDCS) has established a causal link between the right cerebellum and language functions. For example, applying tDCS over the right cerebellum in healthy individuals exerts modulatory effects on various speech/language tasks, such as verbal generation (Pope and Miall, 2012), verbal fluency (Turkeltaub et al., 2016), semantic prediction (D’Mello et al., 2017), and speech motor learning (Lametti et al., 2018). Clinically, several recent studies showed augmentation of SLT in post-stroke aphasia with tDCS over the right cerebellum (Sebastian et al., 2016, 2020; Marangolo et al., 2018). For example, one randomized, double-blind, sham-controlled study found significant improvement in verbal generation in patients with post-stroke aphasia following 4-week cathodal tDCS over the right cerebellum coupled with SLT (Marangolo et al., 2018). In another randomized, double-blind, sham-controlled study (Sebastian et al., 2020), both cathodal and anodal tDCS over the right cerebellum led to improved picture naming in chronic post-stroke aphasia, while greater gains were noted for patients receiving cathodal cerebellar tDCS. Taken together, these studies provide evidence suggesting that the cerebellum may be an optimal site of neuromodulation for the treatment of post-stroke aphasia.

On the other hand, continuous theta burst stimulation (cTBS) is applied over the right cerebellum in the present study protocol. As a specific form of rTMS, cTBS produces an inhibitory effect on cortical excitability for up to 60 min after less than 1-min of stimulation (Huang et al., 2005). In addition to modulating cerebellar activity (Koch, 2010), cTBS over the right cerebellum exerts modulatory effects on speech/language production (Arasanz et al., 2012; Sheu et al., 2019). Cerebellar TBS is safe and tolerable and has reported no serious adverse effects (Hurtado-Puerto et al., 2020). The previous studies have shown that the cerebellar cTBS modulates motor cortical excitability and improves motor symptoms (e.g., gait ataxia and dyskinesia) in a variety of neurodegenerative diseases, such as Parkinson’s disease (PD) and spinocerebellar ataxia (SCA) (Koch et al., 2009, 2014; Benussi et al., 2020; Maas et al., 2020). Moreover, one recent study on patients with SCA showed that single-session c-TBS over the right cerebellum produces facilitatory effects on their abnormalities in auditory-motor integration for vocal pitch regulation (Lin et al., 2022). Therefore, it is plausible to assume that stimulating the right cerebellum with inhibitory cTBS coupled with SLT leads to beneficial effects on chronic post-stroke aphasia, which is yet to be answered.

To this end, the purpose of the present study is to investigate the short- and long-term effectiveness of cTBS over the right cerebellum in augmenting language recovery in chronic post-stroke aphasia in a randomized, sham-controlled design. In addition to aphasia test batteries and speech acoustic analyses for evaluation of treatment outcome, the resting-state functional magnetic resonance (RS-fMRI) is also used to explore the neural mechanisms underlying aphasia rehabilitation. The previous studies have shown associations between improved spelling or phonemic fluency and increased resting-state functional connectivity between the cerebellum and language-related cortical regions for patients with chronic post-stroke aphasia or healthy individuals following tDCS over the right cerebellum (Sebastian et al., 2016; Turkeltaub et al., 2016). Accordingly, we hypothesize that, following cTBS over the right cerebellum, patients with chronic post-stroke aphasia will exhibit improved language functions and increased functional connectivity within the cerebro-cerebellar language network. This randomized, sham-controlled study provides significant new insights into the therapeutic efficacy of cTBS over the right cerebellum to augment SLT for post-stroke aphasia and the underlying neuroplastic mechanisms.

## Methods

### Study Design

**FIGURE 1:**
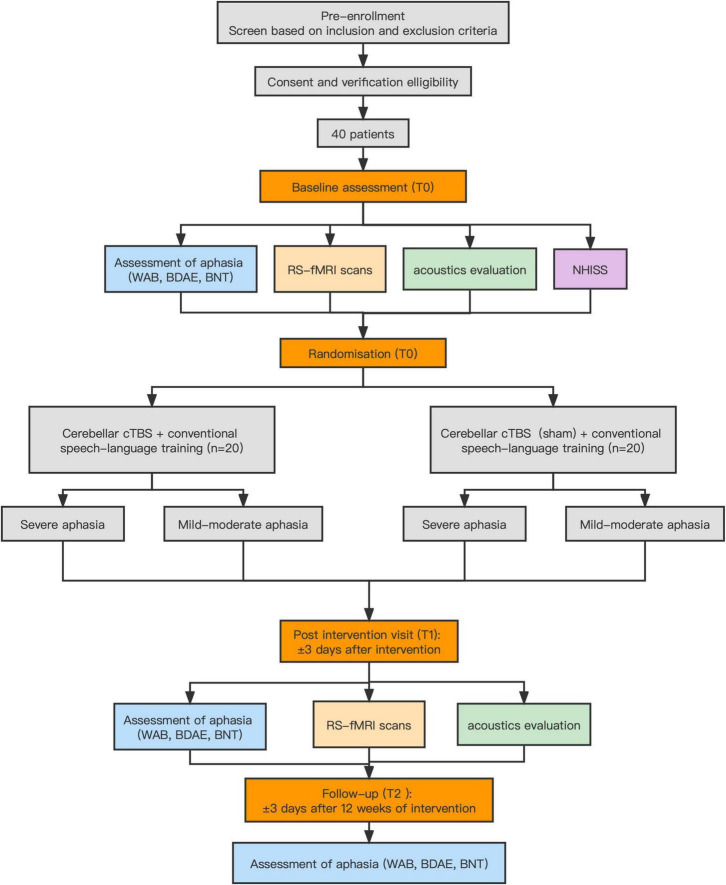
Flow chart of study procedure. cTBS, continuous theta burst stimulation.

This is a prospective, randomized, sham-controlled, single-center study (Registration number: ChiCTR210049828) that will be conducted in the Department of Neurorehabilitation, Wuxi Tongren Rehabilitation Hospital, China. Hospitalized patients with chronic post-stroke aphasia are recruited to participate in the study. They are randomly assigned with a ratio of 1:1 to a real or sham stimulation group. Details about the study design and data collection are shown in Figure 1 and Table 1. On the day of enrollment (T0), after the end of cerebellar cTBS intervention (T1), and 12 weeks post-treatment completion (T2), aphasia test batteries and speech acoustic analyses are performed to evaluate language functions in post-stroke aphasia. Also, RS-fMRI data are collected before and after the end of the intervention. This study has been approved by the Ethics Committee of Wuxi Mental Health Center and Wuxi Tongren Rehabilitation Hospital (WXMHCIRB2021LLky078).

**TABLE 1:** Overview of data collection and study timings.

Study period visits	Pre-enrollment	T0	T1	T2
Relative start of treatment days	−1 week	Day 0	±3 days after intervention	±3 days after 12 weeks of intervention
Screening (in-/exclusion criteria)	×			
Written informed consent	×			
Medical history	×			
Randomization		×		
NIHSS		×		
WAB		×	×	×
BDAE		×	×	×
BNT		×	×	×
Acoustic evaluation		×	×	×
RS-fMRI		×	×	

### Participants

The inclusion and exclusion criteria of the present study correspond to the guidelines for using rTMS in clinics and research (Wassermann, 1998; Rossi et al., 2009; Rossini et al., 2015). Participant inclusion criteria are: (1) patients aged 40–80 years; (2) first-ever unilateral ischemic stroke on the left hemisphere; (3) longer than 6 months post-stroke; (4) non-fluent aphasia as confirmed by the Western Aphasia Battery (WAB); (5) right-handed, native-Chinese speakers without experience of professional vocal or instrumental training; (6) Boston Diagnostic Aphasia Examination (BDAE) Grades I–III; and (7) elementary education level and above, with normal or corrected to normal vision. Participant exclusion criteria are: (1) history of substance or alcohol abuse, premorbid seizures, or neuropsychiatric diseases; (2) contraindications involving TMS and fMRI (e.g., skull defect or skin damage at the stimulation site, intracranial implant, cardiac pacemaker, and implanted drug pumps); (3) history of neurosurgical treatment; (4) unable to cooperate with assessment and treatment due to severe cognitive impairment; and (5) other neurological disease. All participants provide written informed consent prior to participating in the study.

### Sample Size

The sample size was calculated using PASS software (v.15.0) on the basis of previous studies. With an effect size of 0.5 based on the WAB-AQ (Hu et al., 2018), we expect that the target effect size had 80% of power with a type I error of 5% (α = 0.05). Thus, the sample size of each group is at least 16. With an assumption of 20% dropout rate, a sample size of 20 participants will be targeted for each group.

### Randomization and Blinding

Once the inclusion criteria are satisfied, the participant is allocated to one real or sham cTBS group using the network-based random sequence generator to receive real or sham cTBS over the right cerebellum coupled with SLT. Based on the previous studies, patients will receive a 10-day cerebellar cTBS within 2 weeks (Thiel et al., 2013; Wang et al., 2014; Hara and Abo, 2021). The concealment of allocation is performed using sealed envelopes with numbers. The participants and physicians performing the SLT or the data collection/processing are blinded to the grouping. Note that physicians conducting the cTBS intervention cannot be blinded due to the nature of the cTBS intervention and are thus not involved in the research process. The designer and the staff responsible for allocation concealment will not participate in the whole intervention.

### Interventions

The application of cerebellar cTBS is administered using Magneuro 100 (Vishee Medical Technology Co., Ltd., Nanjing, China) equipped with a figure 8-shaped coil (7 cm out diameter). Before intervention, a single-pulse TMS will be applied over the right primary motor cortex to determine active motor threshold (AMT). The AMT is defined as the lowest stimulus intensity that produces a motor evoked potential (MEP) of >200 μV in at least 5–10 consecutive trials with 10% maximal voluntary contraction of the first dorsal interosseous muscle of the non-paretic hand (Koch et al., 2020). Each participant receives real or sham cTBS over the Crus I of the right lateral cerebellum (3 cm right and 1 cm inferior to the inion) at 80% of AMT (Lin et al., 2022). A standard cTBS protocol consists of bursts of 3 pulses at 50 Hz that are repeatedly presented at 5 Hz, resulting in a total of 600 pulses in 40 s. The coil is oriented vertically to the target with the handle pointing superiorly during real stimulation; while the stimulation face of the coil is turned 90°, so that the side of the coil is placed on the target during sham stimulation (Rastogi et al., 2017). Immediately following real or sham cerebellar cTBS, all participants receive a 30-min SLT that includes training on comprehension, expression of spoken language, semantic, and phonological processing. The content of SLT will be modified based evaluation of language function on an individual level. Totally, each participant receives 10 sessions of cerebellar cTBS coupled with SLT (5 sessions per week) within 2 weeks.

### Outcome Measures

The primary outcome measure is the WAB assessment that is divided into four subitems including spontaneous speech, auditory verbal comprehension, repetition, and naming. The original scores of the four items are 20, 200, 100, and 100, respectively. An aphasia quotient (AQ) is calculated using the following formula: AQ = (spontaneous speech + auditory verbal comprehension/20 + repetition/10 + naming/10) × 2. The AQ indicates the severity of aphasia and can be used as an index for evaluating the improvement and deterioration of aphasia. The highest AQ score is 100, and the normal range is 98.4–99.6. AQ <93.8 is considered as aphasia.

In addition to the WAB scores, there are several secondary measures of treatment outcome including BDAE, Boston Naming Test (BNT), speech acoustic parameters, and RS-fMRI. BDAE is used to measure the severity of impaired language function that can be divided into five grades from severe to mild. The BNT is an assessment tool to measure the confrontational word naming of 30 object pictures. The speech acoustic parameters include fundamental frequency, intensity, speaking rate, maximum phonation time, range of voice features, and speech intelligibility. Speech signals are recorded using a laptop at sampling frequency 44.1 K Hz and Praat software is used to extract acoustic parameters.

RS-fMRI data are acquired using a 3.0 T MAGNETOM Skyra scanner (Siemens, Germany). The participants are instructed to lie still with their eyes closed and think of nothing. Functional images are obtained using echo-planar imaging (EPI) pulse sequence with the following parameters: repetition time (TR) = 2,000 ms; echo time (TE) = 30 ms; 35 slices, field of view (FOV) = 224 mm × 224 mm; slice thickness = 3.5 mm; layer spacing 0.7 mm; flip angle (FA) = 90°; acquisition matrix 64 × 64; voxel size = 3.5 mm × 3.5 mm × 3.5 mm. For registration purposes, a set of high-resolution structural images are acquired through a T1-weighted sequence: 192 sagittal slices; slice thickness = 1 mm; TR = 6.6 ms; TE = 3.1 ms; FA = 12°; FOV = 256 mm × 256 mm. The scanning lasted for 8 min and produced 240 brain volumes.

### Data Analysis

Repeated-measures analysis of variances (RM-ANOVAs) are performed to examine differences in the WAB, BDAE, and BNT scores as well as speech parameters across the conditions, with a within-subject factor of phase (pre- vs. post-treatment) and a between-subject factor of group (real vs. sham cTBS). In the *post hoc* analysis, multiple comparisons are corrected with Bonferroni adjustment. Prior to entering the data into the RM-ANOVAs, Kolmogorov–Smirnov test is used to verify whether they are normally distributed. For non-normally distributed measures, the Wilcoxon Signed Rank Test is used for comparison across the conditions. The intention-to-treat (ITT) analysis is used when there are missing data.

The preprocessing of RS-fMRI data is conducted using DPABI software (v. 6.1) on the MATLAB platform (Mathworks, Natick, MA, United States) (Yan et al., 2016). The main steps are as follows: (1) use MRIConvert software (v. 2.1.0, Lewis Center, Eugene, OR, United States) to convert raw data in DICOM format to NIFI format; (2) remove the first 10 slices and correct the remaining 230 slices for slice timing; (3) correct head motion exceeding 3 mm in any direction or head rotation exceeding 3°; (4) spatially normalize the functional images to a standard Montreal Neurological Institute (MNI) space and resample them to a voxel size of 3 mm × 3 mm × 3 mm; (5) apply a band-pass filter (0.01–0.08) for each voxel to reduce the influence of low frequency fluctuation and high-frequency noise (Lv et al., 2018); and (6) smooth the functional data with a 6-mm full width at half maximum (FWHM) Gaussian kernel.

After the preprocessing, regional homogeneity (ReHo), degree centrality (DC), and seed-to-voxel analyses are conducted to measure the local and global functional connectivity within the language networks. ReHo is calculated based on the Kendall’s coefficient of concordance (KCC), which measures the consistency of the time signal between a voxel and surrounding voxels. Higher ReHo values represent better consistency between the local voxel and the regional brain activities. DC values reflect the network connection intensity between a certain voxel and all of the brain, indicating the importance of this voxel as the network node (Zang et al., 2004). One-way ANOVAs are conducted on the ReHo and DC values to explore differences across the conditions within the predefined gray matter mask. The ANOVA F maps are corrected using Gaussian random field (GRF) correction (single voxel *p* < 0.001, cluster level *p* < 0.05). The ReHo and DC values of the peak voxels in the surviving clusters are extracted and entered into SPSS (v. 22) for further analysis. Seed-to-voxel analyses are conducted with *a priori* regions of interest (ROI). The ROIs consist of language-related regions in cerebellar Crus I; IFG pars opercularis; IFG pars triangularis; primary motor cortex (M1); anterior and posterior STG; angular gyrus; anterior and posterior supramarginal gyrus (SMG), which are selected from the AAL atlas (Tzourio-Mazoyer et al., 2002). These ROI-based functional connectivity maps are statistically compared between pre- and post-treatment and between real cTBS and sham stimulation group. Differences across the conditions are considered significant when *p* < 0.05 at the voxel level, with a false discovery rate (FDR) cluster corrected *p* < 0.05.

### Safety

Adverse effects are defined as any negative experiences that occur to the patients who underwent the cerebellar cTBS or MRI scanning. All adverse effects will be reported by the investigator during the treatment and within 1 week after the end of treatment. Particularly, seizures that are the most severe TMS-related adverse effects with a risk of approximately 0.02%, are only expected to occur during or immediately after cerebellar cTBS. In addition, adverse effects that occur during the MRI scanning will be reported within 24 h. Earplugs will be provided to patients to protect their hearing against noise. Any serious incident will be immediately reported to the Medical Research Ethics Committee of Wuxi Tongren Rehabilitation Hospital.

## Discussion

This randomized, sham-controlled clinical trial investigates the neural and behavioral effects of cTBS over the right cerebellum on chronic post-stroke aphasia. We hypothesize that stimulating the right cerebellum with inhibitory cTBS can produce beneficial effects in augmenting SLT for language recovery in post-stroke aphasia by regulating functional connectivity between the right cerebellum and the cerebral cortical regions involved in language processing. These results can provide supportive evidence that the right cerebellum may be a potential optimal stimulation target for the treatment of chronic post-stroke aphasia.

Variability in lesion location and size in brain reorganization for post-stroke aphasia complicate efforts to determine the optimal stimulation strategy at the cortical level (Breining and Sebastian, 2020). Right cerebellar stimulation, in contrast, can potentially serve as a single target site that can be used across patients with post-stroke aphasia with varying site and size of lesion in the left hemisphere (Turkeltaub et al., 2016). The present study proposes a new stimulation protocol for language recovery in chronic post-stroke aphasia by applying inhibitory cTBS over the right cerebellum, since this region has been demonstrated to be essentially involved in a variety of language functions (Gordon, 1996; Desmond and Fiez, 1998; Murdoch, 2010; Stoodley, 2012; Mariën and Borgatti, 2018) and damage to this region leads to impaired language performance (Marien et al., 1996, 2001; Silveri et al., 1998). More importantly, several clinical studies reported improvement in verbal generation or naming functions in the patients with chronic post-stroke aphasia following anodal or cathodal tDCS over the right cerebellum (Sebastian et al., 2016, 2020; Marangolo et al., 2018), providing further evidence in support of the right cerebellum as an optimal stimulation site for neuromodulation in aphasia. On the other hand, Sebastian et al. (2020) found greater beneficial effects on naming functions (relative to sham) when chronic post-stroke patients received cathodal tDCS over the right cerebellum than when they received anodal cerebellar tDCS. And applying inhibitory cTBS over the right cerebellum led to facilitatory effects on impaired vocal motor control in patients with SCA (Lin et al., 2022). As compared to rTMS, cTBS induces robust, long-lasting changes in activation in much shorter periods. Therefore, cTBS over the right cerebellum proposed in the present study protocol may be a promising technique to augment SLT for language recovery in chronic post-stroke aphasia.

The present study protocol also investigates the neural effects of cTBS over the right cerebellum on chronic post-stroke aphasia with RS-fMRI. The majority of previous studies reported behavioral performance assessed by aphasia test batteries following stimulation with rTMS or tDCS (Sebastian et al., 2020), but little is known about the underlying neural mechanisms (Sebastian et al., 2016). In a case report study that combined tDCS and RS-fMRI, Sebastian et al. (2016) found that improvement in spelling induced by anodal tDCS over the right cerebellum was accompanied by increased functional connectivity of the cerebro-cerebellar network. In another study on healthy individuals (Turkeltaub et al., 2016), in addition to improved phonemic fluency, increased resting-state functional connectivity between the cerebellum and speech-motor areas and within the left-lateralized network involved in cognitive aspects of language and motor aspect of speech production were found following tDCS over the right posterolateral cerebellum. Moreover, cerebellar cTBS can modulate activity in the neural pathway that reciprocally links the cerebellum and prefrontal and parietal regions involved in language production (O’Reilly et al., 2010; Stoodley, 2012) by altering short- and long-intracortical inhibition (Koch et al., 2008). Therefore, it is possible that c-TBS over the right cerebellum is effective in an augmenting chronic post-stroke aphasia by altering functional connectivity of the cerebro-cerebellar network.

The present study applies a figure-of-8 coil to the cerebellum. This coil has been shown to be a flat coil model with increased trial tolerances (Hardwick et al., 2014) that can stimulate the superficial layers of the cerebellar cortex (Li et al., 2019). The reported average depth of the lateral cerebellar gray matter is approximately 14.6–14.7 mm from the scalp surface, which is within a range of ∼30 mm at 100% peak output of the figure-of-8 coil (Hardwick et al., 2014). Several studies have successfully applied rTMS or TBS over the cerebellum with a figure-of-8 coil to change motor or speech performance (Theoret et al., 2001; Koch et al., 2008; Popa et al., 2010; Demirtas-Tatlidede et al., 2011; Lin et al., 2022). On the other hand, the present study protocol performs acoustic signal analysis to extract speech parameters for the evaluation of language function with the WAB, BDAE, and BNT scores. The previous research has shown impaired abilities of speech production and motor control in patients with non-fluent aphasia (e.g., Broca’s aphasia) (Hillis, 2007; Behroozmand et al., 2018). Therefore, performing speech acoustic analysis has the potential to comprehensively elucidate the mechanisms underlying the treatment of post-stroke aphasia with cTBS over the right cerebellum coupled with SLT.

## Conclusion

In conclusion, the present study protocol proposes a randomized, sham-controlled clinical trial to investigate the efficacy of cTBS over the right cerebellum in augmenting language recovery in chronic post-stroke aphasia and the neural mechanisms underlying the treatment outcome. If this intervention study produces a significant positive effect as expected, the findings will provide valuable evidence for establishing a novel and feasible stimulation protocol to promote language recovery from chronic post-stroke aphasia.

## Ethics Statement

The studies involving human participants were reviewed and approved by the Medical Research Ethics Committee of Wuxi Mental Health Center (Wuxi Tongren Rehabilitation Hospital). The patients/participants provided their written informed consent to participate in this study.

## Author Contributions

CR and PL designed the study. CR, MC, YS, PL, and KZ drafted the protocol. MC coordinated the trial. FG, HF, YJ, and DS performed data acquisition and clinical evaluation. GH and BS conducted the interventions. CR, YS, and MC revised the manuscript. All authors read and approved the final manuscript.

## Conflict of Interest

The authors declare that the research was conducted in the absence of any commercial or financial relationships that could be construed as a potential conflict of interest.

## Publisher’s Note

All claims expressed in this article are solely those of the authors and do not necessarily represent those of their affiliated organizations, or those of the publisher, the editors and the reviewers. Any product that may be evaluated in this article, or claim that may be made by its manufacturer, is not guaranteed or endorsed by the publisher.
