# Supplementary material for: Cerebellar Continuous Theta Burst Stimulation for Aphasia Rehabilitation: Study Protocol for a Randomized Controlled Trial
Source: Front Aging Neurosci. 2022 Jun 2;14:909733. doi: 10.3389/fnagi.2022.909733 (PMC9201405; doi:10.3389/fnagi.2022.909733)
Supplement: Supplementary file 1 [file Table_1.DOCX]

Supplementary Table 1. Trial characteristics based on WHO trial registration dataset

| Data category | Information |
| --- | --- |
| Primary registry and trial identifying number | ChiCTR210049828 |
| Date of registration in primary registry | 2021.08.10 |
| Secondary identifying numbers | N/A |
| Trial protocol version | Version 1 |
| Source(s) of monetary or material support | National Natural Science Foundation of China (No. 81772439), Natural Science Foundation of Jiangsu Province (No. BK20201138), Wuxi Taihu Talent Project (No. WXTTP2020008), Top Talent Support Program for Young and Middle-aged People of Wuxi Health Committee and General Project from Wuxi Health Commission (No. MS201911) |
| Primary sponsor | N/A |
| Secondary sponsor | N/A |
| Contact for public queries | CLR, rencaili@njmu.edu.cn |
| Contact for scientific queries | CLR, rencaili@njmu.edu.cn |
| Public title | Impact of cerebellar theta burst stimulation combined with speech therapy on language performance in patients with chronic aphasic after stroke: a randomised controlled trial |
| Scientific title | Impact of cerebellar theta burst stimulation combined with speech therapy on language performance in patients with chronic aphasic after stroke: a randomised controlled trial |
| Countries of recruitment | China |
| Health condition(s) or problem(s) studied | Stroke with aphasia. |
| Intervention(s) | Active comparator: cTBS stimulation (application of 600 pulses with a frequency of 50 Hz, in a theta-rhythm of 5 Hz for 40 s) applied to the right CrusI positioned tangentially to the scalp combined with standard speech-language therapy.  Sham comparator: Sham stimulus applied to the right CrusI positioned vertically to the scalp combined with standard speech-language therapy. |
| Key inclusion and exclusion criteria | Inclusion criteria: (1) patients age 25–75 years; (2) first-ever unilateral ischemic stroke on the left; (3) admission over six months after stroke onset; (4) patients with nonfluent aphasia assessed by Western Aphasia Battery (WAB); (5) right-handed, native Chinese, normal speech function prior to the onset, no professional vocal or instrumental training; (7) Boston Diagnostic Aphasia Examination (BDAE) Grades Ⅰ–Ⅲ; (8) elementary education and above, with normal vision and hearing; (6) written informed consent.  Exclusion criteria: (1) history of substance or alcohol abuse, history of premorbid seizures or neuropsychiatric diseases, (2) pacemakers in the body or metal implants in the brain, ears or other parts of the body; (3) skull defect or skin damage at the stimulation site |
| Study type | Interventional  Allocation: concealed randomisation  Masking: double-blind (subject, assessor).  Assignment: parallel  Primary purpose: prevention and improvement |
| Date of first enrolment | October, 2021 |
| Target sample size | 40 |
| Recruitment status | Recruiting |
| Primary outcome(s) | improvement in the WAB scores |
| Key secondary outcomes | BDAE, the Boston Naming Test (BNT), acoustic index, regional homogeneity (ReHo), degree centrality (DC) and seed-to-voxel analyses from RS–fMRI |
